# Identification of potential therapeutic targets for systemic lupus erythematosus based on GEO database analysis and Mendelian randomization analysis

**DOI:** 10.3389/fgene.2024.1454486

**Published:** 2024-10-16

**Authors:** Aishanjiang Apaer, Yanyan Shi, Alimijiang Aobulitalifu, Fujie Wen, Adalaiti Muhetaer, Nuermaimaiti Ajimu, Maierhaba Sulitan, Lei Cheng

**Affiliations:** ^1^ Department of Pharmacy, The First People’s Hospital of Kashi Prefecture, Xinjiang, China; ^2^ Department of Pediatrics, The First People’s Hospital of Kashi Prefecture, Xinjiang, China; ^3^ Department of Anesthesiology, The First People’s Hospital of Kashi Prefecture, Xinjiang, China

**Keywords:** systemic lupus erythematosus (SLE), GEO database, WGCNA, Mendelian randomization analysis, molecular docking

## Abstract

**Background:**

Systemic lupus erythematosus (SLE) is a complex autoimmune disease. Current treatments mainly rely on immunosuppressants, which lack specificity and pose challenges during treatment. This study aims to deeply explore the molecular pathogenic mechanism of SLE through gene expression databases (GEO) and bioinformatics analysis methods, combined with Mendelian randomization analysis, to provide key clues for new therapeutic targets.

**Methods:**

In this study, the SLE-related gene chip dataset GSE65391 was selected from the GEO database, and the data were preprocessed and statistically analyzed using R language and bioinformatics tools. Differential expression analysis, weighted gene co-expression network analysis (WGCNA), GO, and KEGG enrichment analysis were used to screen differentially expressed genes (DEGs) for functional annotation and pathway localization. Furthermore, Mendelian randomization analysis was conducted to identify core genes closely related to SLE risk, and immune cell infiltration analysis and compound molecular docking studies were performed on the core gene ISG15.

**Results:**

The study successfully screened 3,456 DEGs and identified core gene modules highly related to SLE through WGCNA analysis, including key genes closely related to the pathogenesis of SLE, such as STAT1, DDX58, ISG15, IRF7, and IFIH1. In particular, this study found a significant positive correlation between the ISG15 gene and SLE, suggesting that it may be a potential risk factor for SLE. Additionally, through molecular docking technology, it was discovered that the ISG15 gene can effectively bind to two compounds, genistein, and flavopiridol, which have anti-inflammatory and immunosuppressive effects, respectively. This provides new potential drug targets for SLE treatment.

**Discussion:**

As an immunomodulatory cytokine, ISG15 plays a crucial role in the pathogenesis of SLE. This study found that variations in the ISG15 gene may increase the risk of SLE and exacerbate inflammatory responses and tissue damage through multiple mechanisms. Furthermore, molecular docking revealed that genistein and flavopiridol can effectively bind to ISG15, offering a new approach for SLE treatment. These two compounds, with their anti-inflammatory and immunosuppressive properties, have the potential to slow the progression of SLE by influencing the expression and function of ISG15.

**Conclusion:**

Through comprehensive bioinformatics analysis and Mendelian randomization analysis, this study deeply explored the molecular pathogenic mechanism of SLE and successfully identified ISG15 as a potential therapeutic target for SLE. Simultaneously, molecular docking technology revealed that two compounds, genistein and flavopiridol, have potential therapeutic effects with ISG15, providing new potential drugs for SLE treatment. These discoveries not only enhance our understanding of the pathogenesis of SLE but also provide important clues for developing new treatment strategies.

## Introduction

**FIGURE 1:**
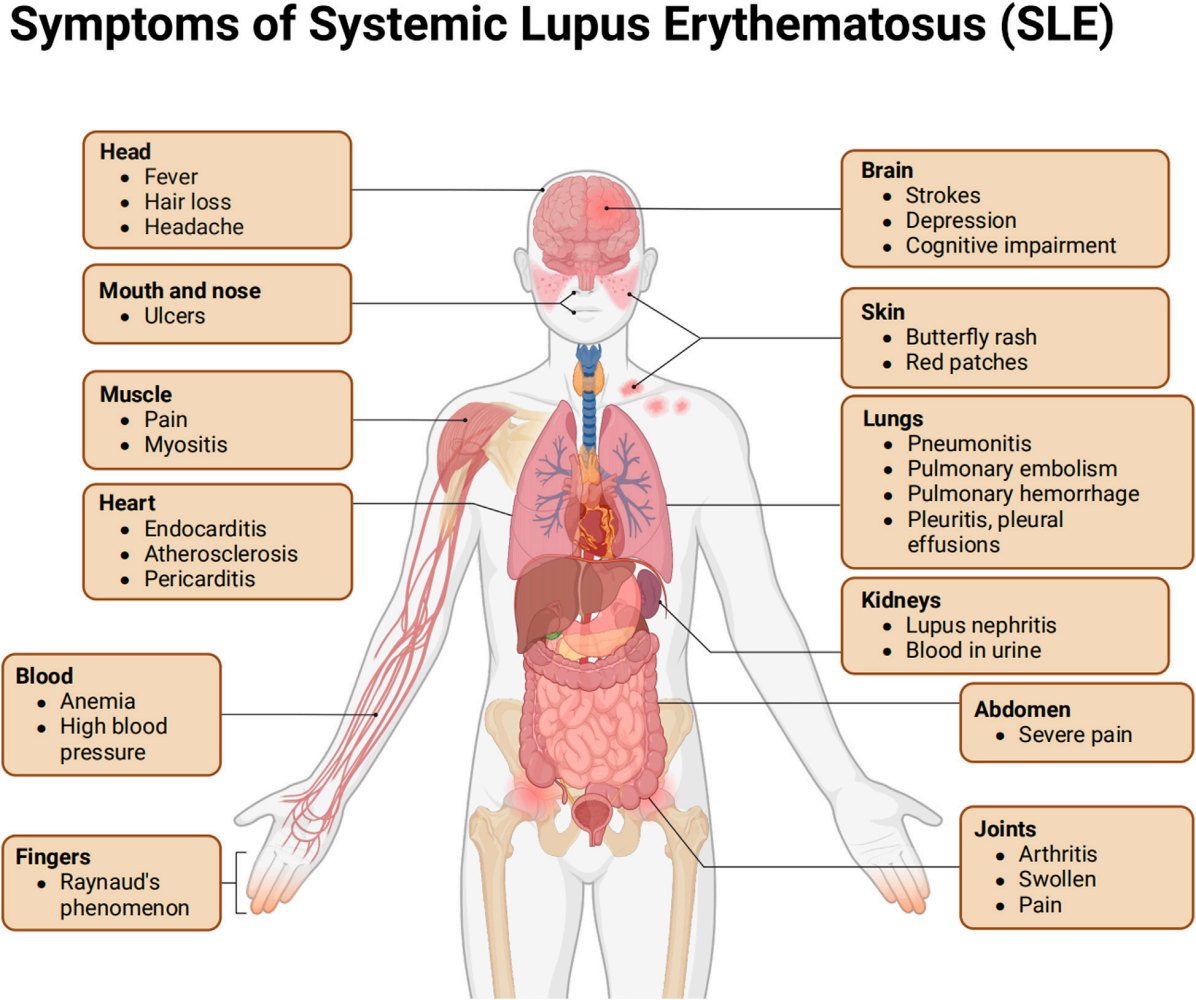
Symptoms of systemic lupus erythematosus (SLE).

Systemic lupus erythematosus (SLE) is a complex autoimmune disease characterized by the loss of self-tolerance and the formation of autoantibodies and immune complexes. These processes further trigger inflammatory responses in multiple organs. The clinical manifestations of SLE are extremely diverse, potentially affecting various parts of the body such as the skin, kidneys, joints, and nervous system (Figure 1). The course of the disease often exhibits chronic or relapsing-remitting patterns. The core pathological mechanism of SLE lies in the body producing antibodies targeting its own antigens. These antibodies bind to antigens, forming immune complexes that deposit in blood vessels and activate intense inflammatory reactions, ultimately leading to widespread tissue damage and organ dysfunction (Durcan et al., 2019). In recent years, the incidence and prevalence of SLE have been on the rise globally. According to statistics, about 3.4 million people worldwide have been diagnosed with SLE (Siegel and Sammaritano, 2024). However, this number may still underestimate the actual number of patients, as many may not be diagnosed promptly in the early stages of the disease. Additionally, there are significant differences in the incidence of SLE across different geographical regions, with an overall incidence rate ranging from 0.3 to 23.2 cases per 100,000 person-years (Accapezzato et al., 2023).

Currently, immunosuppressants are the primary means of SLE treatment. However, these drugs often lack specificity, and due to the unpredictable flare-ups and remissions characteristic of SLE’s course, the treatment process becomes quite challenging (Morand et al., 2023). Therefore, it is particularly important to deeply explore the molecular pathogenic mechanism of SLE, which not only helps us to more accurately diagnose the disease and determine the disease stage, but also may provide key clues for the development of new therapeutic targets.

**FIGURE 2:**
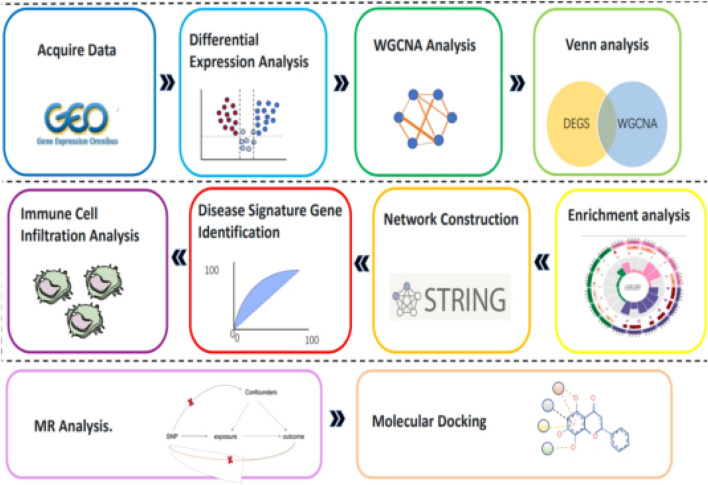
Flowchart.

With the rapid development of high-throughput sequencing technology, we now have the ability to obtain massive amounts of genomics data. By combining bioinformatics analysis methods, these data can be deeply mined to reveal the genetic basis and molecular mechanisms of diseases (Ang et al., 2019). Based on this concept, this study used the gene chip datasets related to SLE in the Gene Expression Omnibus (GEO) of the National Center for Biotechnology Information (NCBI). Through a series of bioinformatics analysis and R software for data preprocessing and statistical analysis, genes with significant differential expression between SLE patients and healthy controls were screened out. Subsequently, GO enrichment analysis and KEGG signaling pathway analysis were used to functionally annotate and locate these differentially expressed genes. To more intuitively show the interaction between genes, we also used Cytoscape software to construct a gene interaction network and identify key genes from it. In addition, we further identified the core genes closely related to the risk of SLE through Mendelian randomization analysis, analyzed the immune cell infiltration of these genes, and docked the core genes with compound molecules (Figure 2).

The aim of this study is not only to reveal the pathogenic mechanism of SLE from the genetic and genomic level, but also to provide new ideas and methods for the diagnosis and treatment of SLE through these findings. We hope that by delving into these key genes and signaling pathways, we can discover new diagnostic markers and develop new therapeutic targets, thereby improving the prognosis and quality of life of SLE patients.

## Materials and methods

### Data acquisition and pre-processing

In this study, we searched the GEO database (https://www.ncbi.nlm.nih.gov/geo/) using the keyword “systemic lupus erythematosus” to identify eligible datasets. We set specific search criteria: “Entry type” was set to “Series,” “Study type” was set to “Expression profiling by array,” the species was limited to humans, and the dataset must include a control group with a sample size of no less than 10 in each group. After careful screening, we ultimately selected the GSE65391 dataset and downloaded its corresponding platform file GPL10558. This dataset includes whole blood transcriptome data from 924 experimental group samples and 24 control group samples. For subsequent bioinformatics analysis, we systematically organized the matrix data files using Perl scripts. Additionally, we successfully converted gene probe IDs to gene symbols (Gene symbol) with the help of the platform file, facilitating subsequent data analysis and interpretation.

### Differential expression analysis

Differential gene expression analysis was performed using Limma, with genes screened based on the criteria of |log2FC| ≥ 0 and adjusted *P*-value <0.05. Volcano plots were generated using ggplot2 to visually demonstrate upregulated and downregulated genes, while Heatmaps were generated to illustrate gene expression patterns, which will support subsequent research.

### Weighted gene co-expression network analysis (WGCNA) screening for signature and co-expressed genes

Using the wgcna software package in R, a weighted gene co-expression network analysis was conducted on the gene expression matrix. Through WGCNA, we clustered genes into different modules and selected the module with the highest correlation to SLE. Core genes of the module were screened based on gene significance (GS) greater than 0.5 and gene-module membership (MM) greater than 0.8. Finally, a Venn diagram was created using the VennDiagram software package to demonstrate the overlap between differentially expressed genes and the core genes identified by WGCNA.

### Enrichment analysis

To explore the signaling pathways significantly enriched by differentially expressed genes, we imported the selected differentially expressed genes into R software. Utilizing the org. Hs.e.g.,.db, ggplot2, and clusterProfiler packages, we employed enrichGO and enrichKEGG algorithms to perform Gene Ontology (GO) and Kyoto Encyclopedia of Genes and Genomes (KEGG) signaling pathway analyses on the genes. This allowed us to gain a deeper understanding of the roles of these genes in biological processes.

### Network construction and identification of key genes

The selected differentially expressed genes (DEGs) were imported into the STRING online database, and a protein-protein interaction (PPI) network was constructed with a confidence level set at 0.4. Relevant data were downloaded for subsequent analysis. Afterwards, the PPI data were imported into Cytoscape 3.9.1 software, and the cytoHubba plugin was utilized to screen out the core genes in the network. The top 10 genes ranked by their Degree were identified as key genes.

### Identification and validation of disease signature genes

We utilized the glmnet package in R to perform gene selection through regularization methods such as Lasso regression, aiming to identify genes critical for disease diagnosis. With the help of the pROC package, we constructed ROC curves to evaluate the effectiveness of these genes in distinguishing between diseased and healthy states, ensuring their practical application value. Furthermore, we used the rms and rmda packages to validate the selected key genes. By constructing nomograms, we visualized the relationship between genes and target events. Through model fitting, calibration, and validation, we assessed the predictive accuracy of the model and the association between genes and diseases.

### Level of immune cell infiltration

Using the CiberSort algorithm, we accurately calculated the relative abundance of various immune cells in the microarray expression profiles and conducted an in-depth analysis of immune cell changes in SLE patients. By comparing the immune cell distribution between healthy individuals and SLE patients, we observed significant differences in immune cells of SLE patients. This discovery provides important clues for further exploring the pathogenesis of SLE.

### MR analysis between signature genes and SLE

We employed a two-sample Mendelian randomization (MR) analysis to investigate the causal relationship between characteristic genes and the risk of systemic lupus erythematosus (SLE), using single nucleotide polymorphisms (SNPs) as instrumental variables. The data was sourced from the Integrated Epidemiological Unit (IEU) OpenGWAS database. Utilizing the “TwoSampleMR” software package and the inverse variance weighting (IVW) method, we evaluated the association between characteristic genes and SLE risk. To ensure the reliability of our findings, we tested for heterogeneity using Cochran’s Q test, and examined potential horizontal pleiotropy using MR-Egger regression and MR-PRESSO analysis. These statistical tests helped confirm the robustness of our results and provided strong evidence for a causal relationship between characteristic genes and SLE risk.

### Molecular docking

We uploaded the core genes to the Comparative Toxicogenomics Database (CTD) (http://ctdbase.org/) to search for active pharmaceutical ingredients related to these genes. Chemical components were then screened based on the number of literature citations (≥5) supporting their correlation. We downloaded verified 3D crystal structures of the core gene targets from the PDB database (https://www.rcsb.org) and the core active ingredient files from the PubChem database. Autodock Vina software was used to perform molecular docking between the core active ingredients and the core gene targets, with binding energy used as an indicator to determine whether the components can bind to the gene targets. Finally, we visualized the molecular docking results using Pymol software.

## Results

### Differentially expressed gene analysis

**FIGURE 3:**
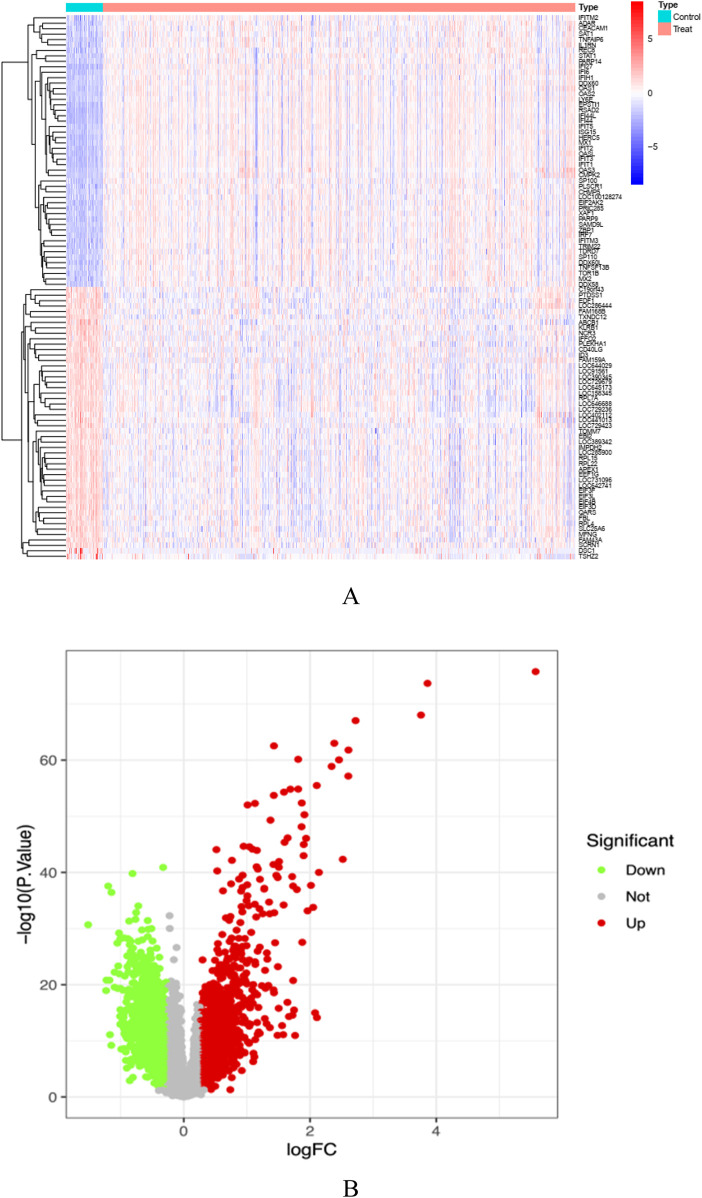
**(A)** Differential gene expression heatmap **(B)** Volcano plo.

In this study, the R language Limma package was used to analyze the dataset. Using the criteria of |log2FC| ≥ 0.58 and adjusted *P* < 0.05, we successfully screened 3,456 differentially expressed genes (DEGs), including 1,626 upregulated genes and 1830 downregulated genes (Figures 3A, B). This discovery provides important clues for further investigation into the mechanisms of the disease.

### WGCNA screening module core genes

**FIGURE 4:**
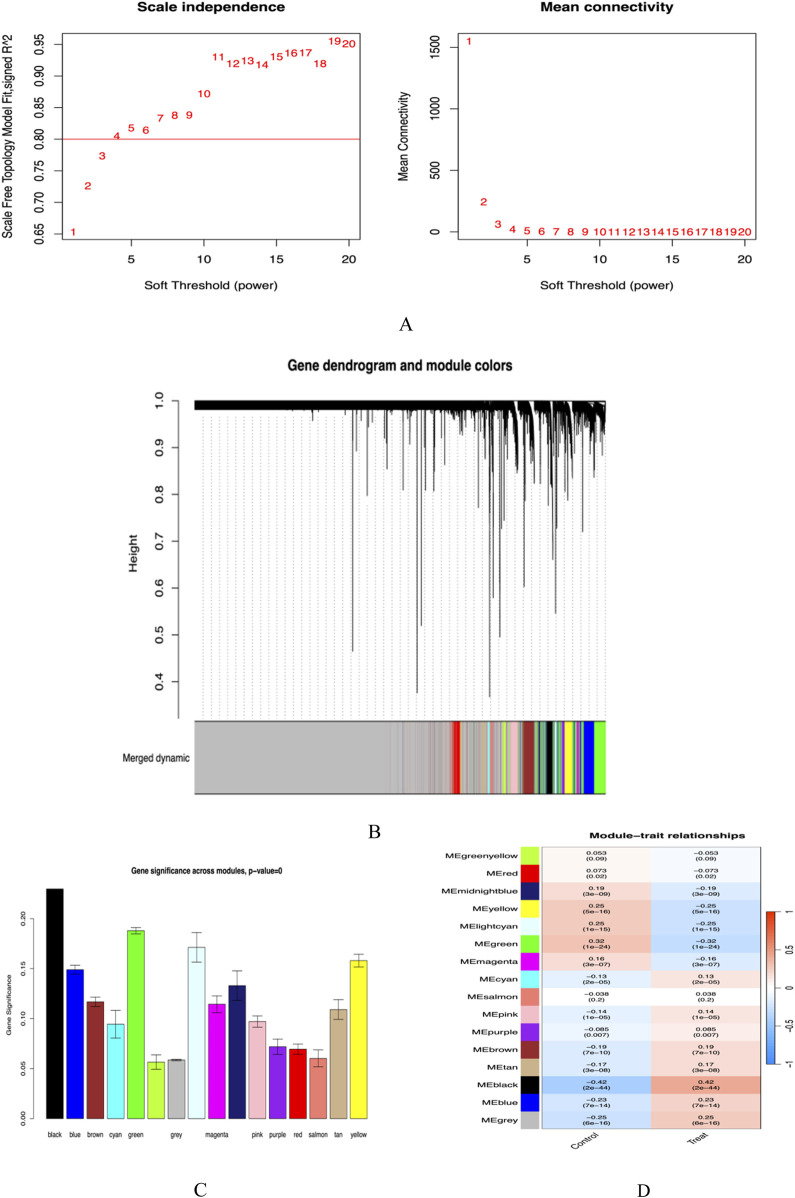
**(A)** Soft threshold screening plot; **(B)** Gene clustering dendrogram; **(C)** Cross-module gene significance plot; **(D)** Module-trait correlation heatmap.

In this study, we utilized the WGCNA method, setting the scale-free fitting index R2 to 0.8 to ensure the effectiveness of network fitting. When the optimal soft threshold power was determined to be 5, R2 reached the criterion of 0.8 (Figure 4A). Through the analysis of the GSE65391 database, we successfully identified 16 gene modules and created a correlation heatmap between diseases and modules using the Spearman correlation coefficient. The results indicated that the “black” module showed the highest correlation with SLE, encompassing 413 genes (Figures 4B–D).

### Venny analysis

**FIGURE 5:**
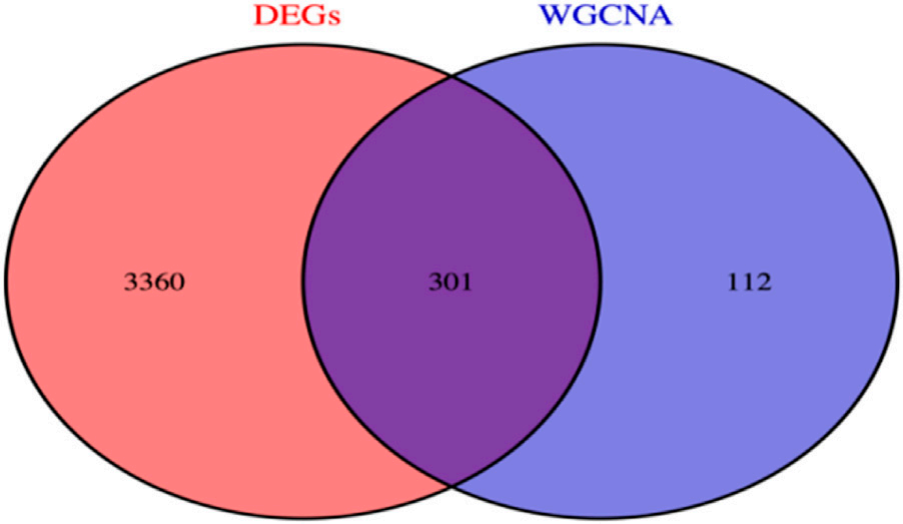
Co-expressed genes.

By using R language, we successfully screened out a total of 301 co-expressed genes between the hub genes of the WGCNA module and differentially expressed genes (DEGs) (Figure 5).

### GO function and KEGG enrichment analysis results

**FIGURE 6:**
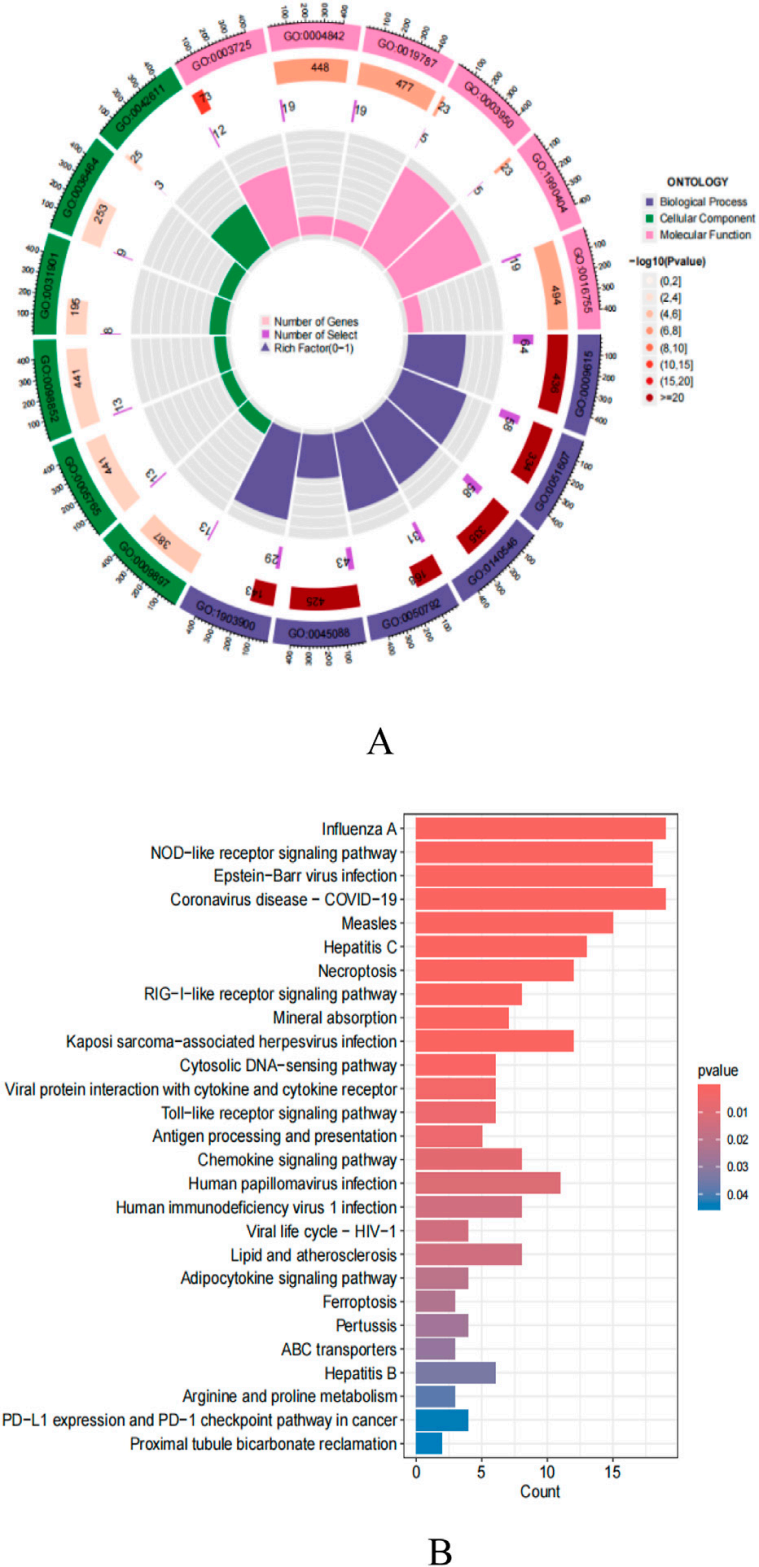
**(A)** GO enrichment analysis; **(B)** KEGG pathway enrichment analysis.

In this study, 301 intersecting target data were imported into R4.3.2 software for detailed GO analysis and KEGG pathway analysis (Figures 6A, B). In the GO analysis, the biological process (BP) category mainly involves critical biological processes such as viral response, response to exogenous stimuli, symbiont defense response, and regulation of innate immune response. The cellular component (CC) analysis revealed the importance of cellular structures such as the outer side of the plasma membrane and lysosomal membrane. The molecular function (MF) focuses on key molecular functions such as double-stranded RNA binding ability, ubiquitin-protein transferase activity, and aminoacyl-tRNA transferase activity. In addition, KEGG pathway analysis showed that these targets are mainly enriched in key signaling pathways such as Influenza A, NOD-like receptor signaling pathway, Epstein-Barr virus infection, necroptosis, RIG-I-like receptor signaling pathway, and Toll-like receptor signaling pathway.

### Interaction networks and core genes of differential gene-encoded proteins

**FIGURE 7:**
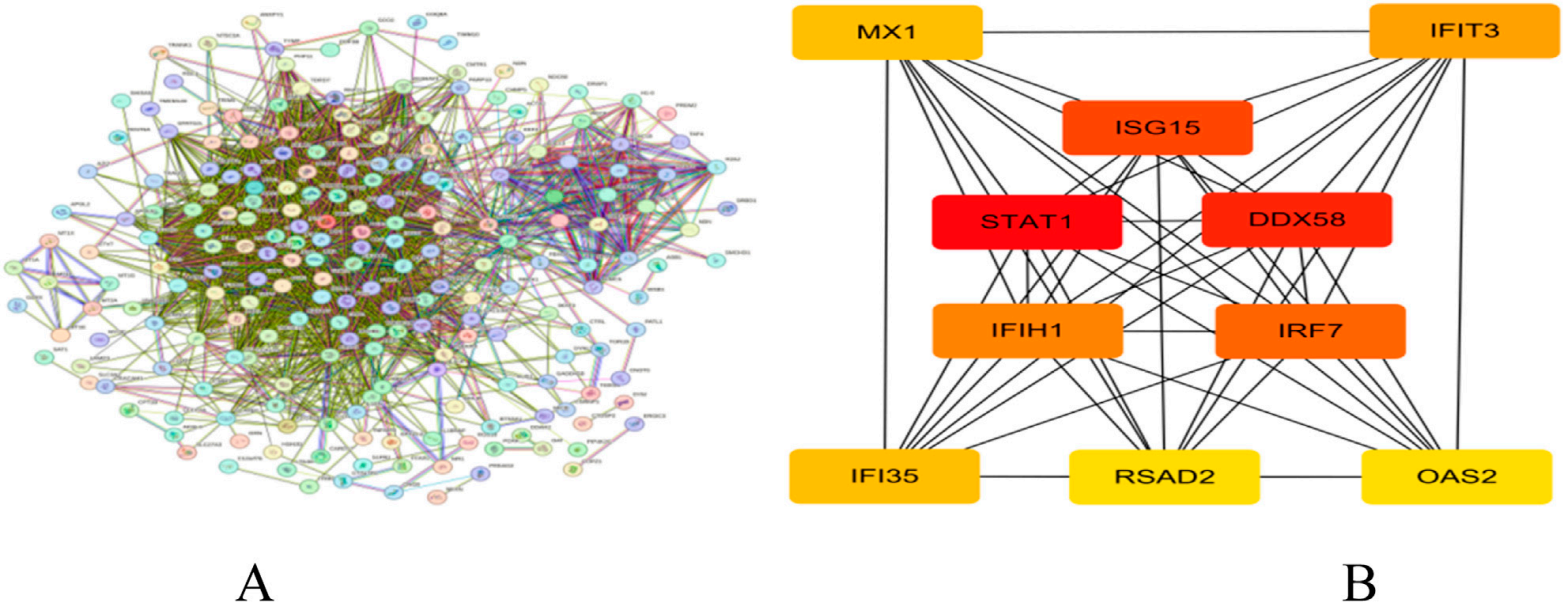
**(A)** PPI network; **(B)** Core gene network and ranking.

We constructed a PPI network for target genes using the STRING online database and imported the obtained tsv format data into Cytoscape software. Through the CytoHubba plug-in and Degree algorithm, we successfully screened out key genes, with their connectivity ranked from high to low as follows: STAT1, DDX58, ISG15, IRF7, and IFIH1 (Figure 7A, B).

### Core gene validation

**FIGURE 8:**
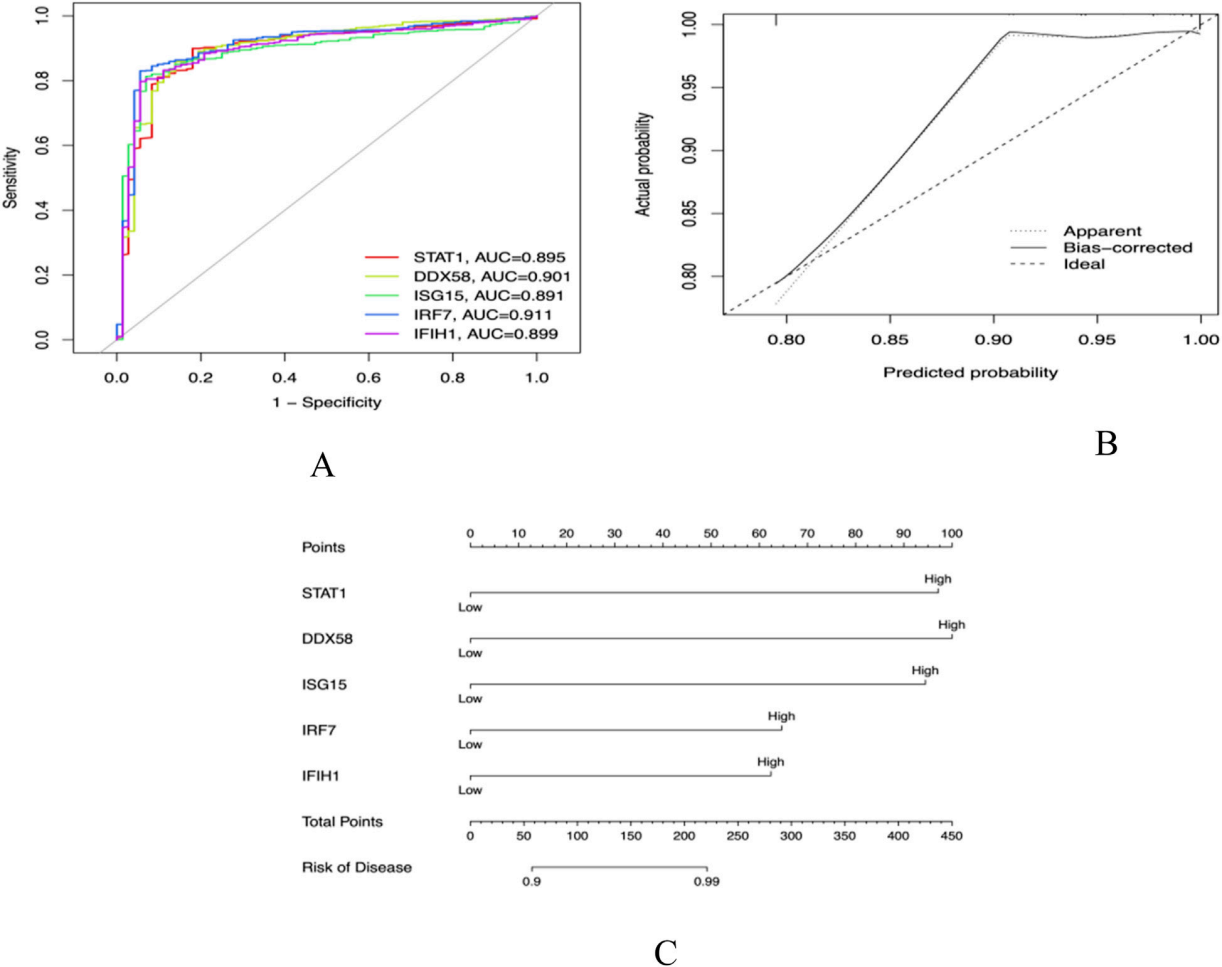
**(A)** Receiver operator characteristic (ROC) curve evaluating the predictive accuracy of nomogram **(B)** Nomogram for predicting **(C)** Decision curve analysis (DCA).

The five core genes, STAT1, DDX58, ISG15, IRF7, and IFIH1, have high diagnostic value for distinguishing between SLE and control samples. Their AUCs are 0.895, 0.9, 0.891, 0.911, and 0.899, respectively, all exceeding 0.75 (Figure 8A), demonstrating good sensitivity and specificity, which further proves the reliability of the above core genes as target genes. By comparing the actual events with the probabilities predicted by the nomogram, our model exhibits good predictive performance (Figures 8B, C).

### The role of immune cells in SLE

**FIGURE 9:**
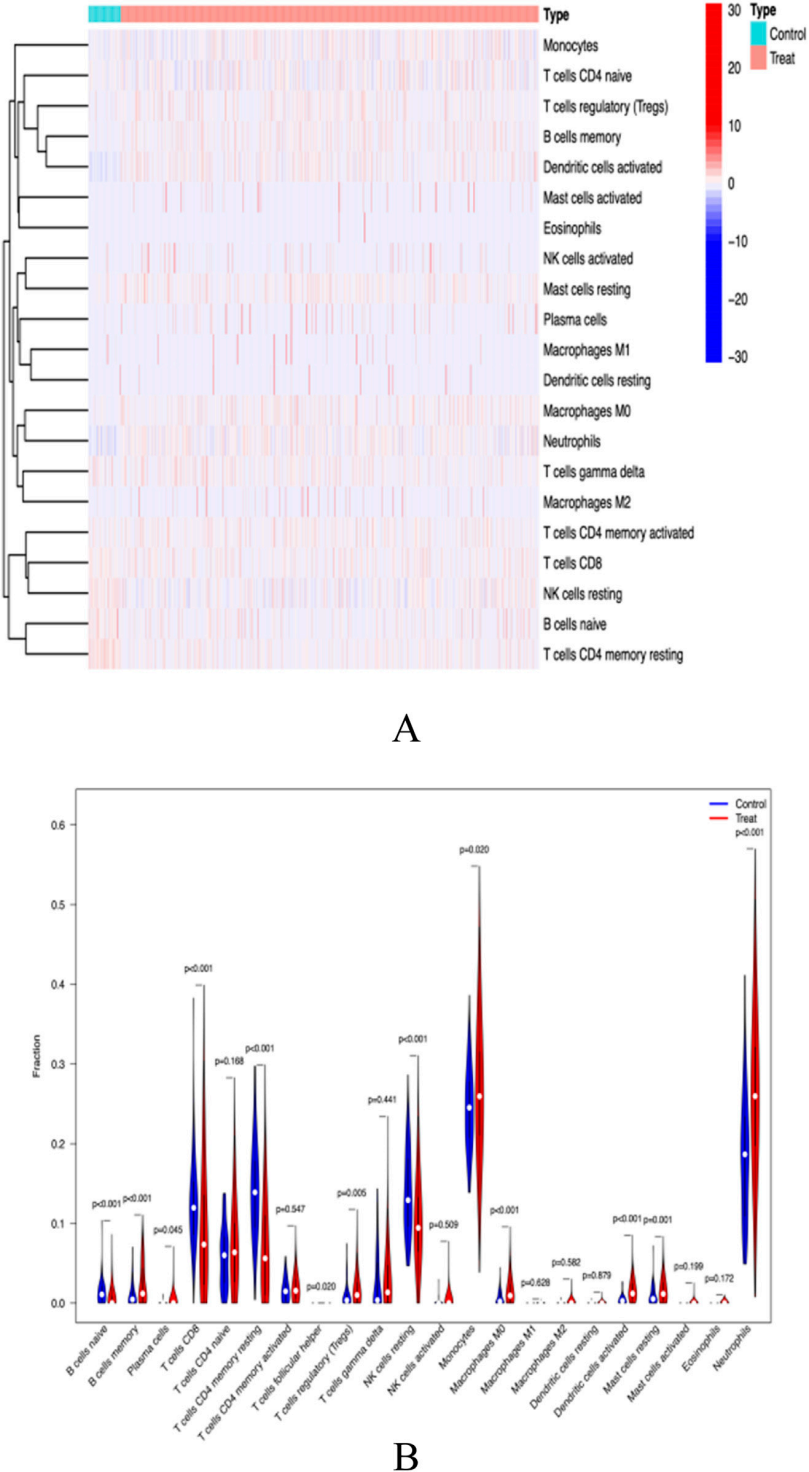
**(A)** immunocyte thermogram **(B)** Immune cell differential analysis.

Functional and pathway analysis of co-expressed genes in SLE indicated a close correlation between SLE and inflammatory and immune processes. The CiberSort algorithm was employed to infer immune cell characteristics and explore the association between co-expressed genes in SLE and immune cell infiltration. The proportions of 21 types of immune cells in each sample are shown (Figure 9A). Significant differences were observed in naive B cells, memory B cells, CD8^+^ T cells, memory CD4^+^ T cells, resting NK cells, and neutrophils between SLE and control samples (Figure 9B).

### Results of Mendelian randomisation analysis of trait genes and SLE

**FIGURE 10:**
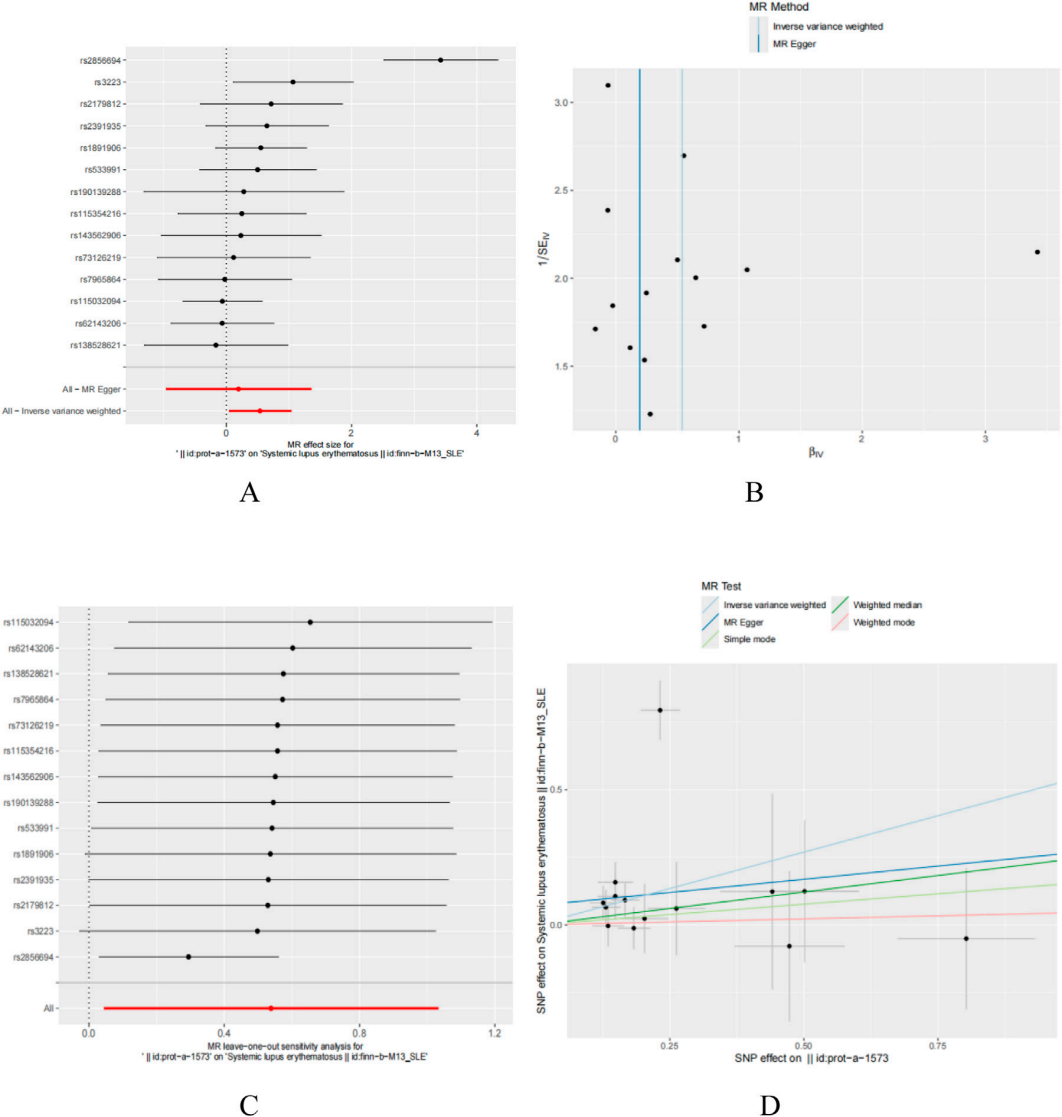
T wo sample Mendelian randomization study results; **(A)** Forest plot showcasing the causal effect of individual SNPs on the SLE; **(B)** Funnel plots utilized to assess the overall heterogeneity of MR estimates for the influence of ISG15 on SLE; **(C)** Forest plot showcasing the causal effect of individual SNPs on the SLE; **(D)** Leave-one-out plot employed to visualize the causal effect of ISG15 on SLE risk when excluding one SNP at a time.

In this study, we employed Mendelian randomization to investigate the potential causal relationship between five genes, namely, DDX58, IFIH1, IRF7, STAT1, and ISG15, and systemic lupus erythematosus (SLE). Due to the lack of research information on IFIH1 and IRF7 in the GWAS database, they were not analyzed. Regarding DDX58 and STAT1, our analysis did not reveal a significant association with SLE. However, for the ISG15 gene, we found a significant positive correlation with SLE. The results of Egger’s test indicated that variations in the ISG15 gene might increase the risk of SLE. Specifically, the Inverse Variance Weighted method yielded statistically significant results (*P* < 0.05), while other methods did not reach statistical significance (Figures 10A–D). In summary, our study suggests that the ISG15 gene may be a potential risk factor for SLE, providing a new perspective on the pathogenesis of SLE.

### ISG15 gene and immune cell penetration

**FIGURE 11:**
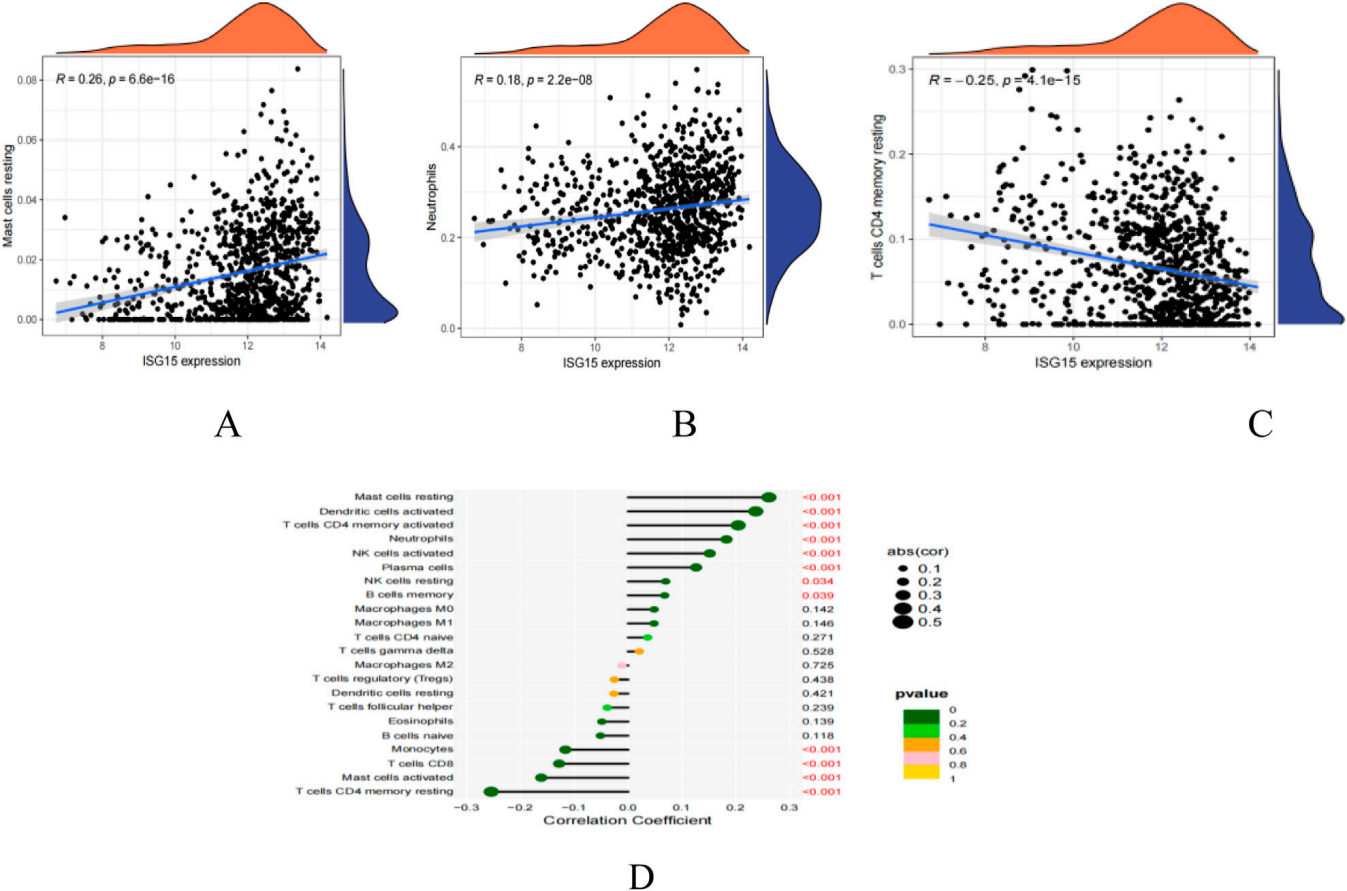
ISG15 is related to immunocyte infiltration levels. **(A)**: Correlation map illustrating the relationship between ISG15 expression and Mast cells restin. **(B)**: Correlation map illustrating the relationship between ISG15 expression and Neutrophils. **(C)**: Correlation map illustrating the relationship between ISG15 expression and T cells CD4 memory resting. **(D)**: Lollipop plot visualizing the correlation between ISG15 and immune cells. Text highlighted in red indicates *p* values that are less than 0.05, denoting statistical significance.

Using CiberSort, statistically significant differences were revealed in Mast cells resting, Neutrophils and T cells CD4 memory resting (Figures 11A–D). This finding suggests a correlation between ISG15 expression and immune cell infiltration, implying that ISG15 may play a role in regulating immune cells in SLE patients.

### Molecular docking of ISG15 gene with compounds

**FIGURE 12:**
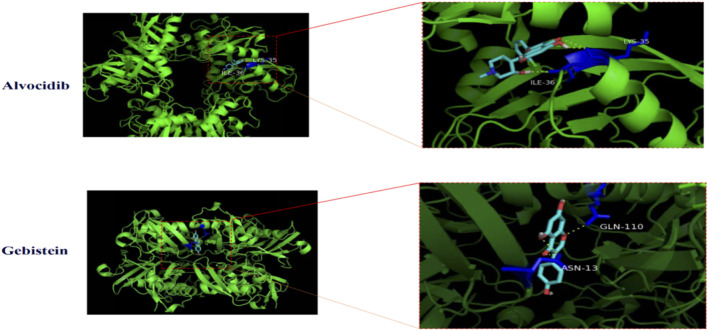
Molecular docking analysis: Alvocidib and Gebistein was docked with ISG15.

The core gene ISG15 was imported into the Comparative Toxicogenomics Database (CTD), and through screening conditions, four pharmaceutically active compounds were identified: Estradiol, Tretinoin, Genistein, and Alvocidib. Relevant files were downloaded from the database, followed by molecular docking using Vina. Compounds with a binding energy of −7 kj·mol^−1^ or lower were considered as candidate drugs. Ultimately, Alvocidib with a binding energy of −8.2 kj·mol^−1^ and Genistein with a binding energy of −7.8 kj·mol^−1^ were selected as potential therapeutic chemicals (Table 1). The molecular docking results were also visualized (Figure 12).

**TABLE1:** Chemical drugs with therapeutic potential.

Target name	PDB ID	Compound name	Pubchem CID	Binding energy (kj·mol^−1^)
ISG15	7S6P	alvocidib	5287969	−8.2
		genistein	5280961	−7.8

## Discussion

Systemic lupus erythematosus (SLE) is a complex autoimmune disease, and its pathogenesis involves multiple factors, including genetics, the environment, and immune system abnormalities (Bakshi et al., 2018). This disease, which is currently incurable, significantly affects patients’ quality of life and poses major challenges for clinical treatment. The aim of this study is to provide a new understanding of the molecular mechanisms of SLE and explore potential targets for future treatment strategies. This will be achieved through comprehensive analysis of SLE-related datasets from the GEO database, application of Mendelian randomization analysis, evaluation of immune cell infiltration, and molecular docking with compounds.

Studies have shown that free ISG15 can serve as an immunomodulatory cytokine (Mirzalieva et al., 2022). This study found significant differences in immune cell abundance between SLE patients and the control group, particularly in the infiltration levels of neutrophils, resting mast cells, resting CD4 memory T cells, and activated natural killer cells, which were higher in SLE patients. The abnormal activation of these immune cells is closely related to the pathogenesis of SLE. An increase in the number of neutrophils can induce plasmacytoid dendritic cells (PDCs) to produce interferon, thereby promoting disease progression (Chasset and Arnaud, 2018). It is worth noting that ISG15 in neutrophils may also induce the production of Th1 lymphocytes with proinflammatory properties, further exacerbating the inflammatory response (Carrillo-Vázquez et al., 2020). A recent study has shown that neutrophil extracellular traps in SLE patients are characterized by the expression of ISG15 (Carrillo-Vázquez et al., 2020). Therefore, ISG15 may act as a persistent proinflammatory stimulus in the pathogenesis of lupus.

Studies have found that ISGylation can regulate mitochondrial function, including basic processes such as respiration and mitosis, and affect the innate immune signal of macrophages (Baldanta et al., 2017). In SLE patients, abnormal expression of ISG15 may lead to mitochondrial dysfunction, thereby affecting the normal function of immune cells (Juncker et al., 2021). In addition, ISG15 is also involved in the DNA damage response (DDR), which is an important factor leading to various diseases (Kang et al., 2022). In SLE, abnormalities in DDR may be related to abnormal expression of ISG15, further exacerbating the progression of the disease.

Research has shown that type I interferons, especially interferon-α, play a central role in the pathogenesis of systemic lupus erythematosus (SLE). In SLE patients, the level of interferon-I is significantly elevated, including immune complexes containing host nucleic acids. Plasmacytoid dendritic cells (PDCs) have been identified as the main cells that produce interferon-I. In addition, there is extensive interaction between PDCs and other immune cells, and this crosstalk jointly drives the continuous production of interferon-I (Gallucci et al., 2021). In addition to PDC, a variety of factors have been reported to induce interferon production, including African ancestry, ultraviolet radiation, infection, specific drugs, and estrogens (Eloranta and Rönnblom, 2016). Interferon-I not only has a direct antiviral effect, but also has a profound impact on the innate and adaptive immunity of SLE patients, specifically promoting the proliferation, differentiation, maturation, or apoptosis of immune cells, and stimulating B lymphocytes to produce autoantibodies (Rönnblom and Leonard, 2019). However, the continuous production of interferon-I can lead to abnormal autoimmune responses and chronic inflammation, ultimately causing tissue damage (Luo et al., 2016).

In addition, studies have also found that ISG15 is associated with an enhanced type I interferon response and promotes the synthesis of interferon-γ (IFNG) by T and B cells (Xiao et al., 2022). In SLE patients, neutrophil extracellular traps are characterized by the expression of ISG15, which further confirms that ISG15 may act as a persistent proinflammatory stimulus in the pathogenesis of lupus. Meanwhile, extracellular ISG15 also exhibits the characteristics of cytokine-like proteins, which can bridge early innate immune responses and IFN-γ-dependent immune responses (Swaim et al., 2020). These findings provide new clues for a deeper understanding of the pathogenesis of SLE.

Under normal physiological conditions, the expression level of ISG15 is minimal. However, in SLE patients, abnormally elevated levels of free ISG15 and ISGylation may contribute to the occurrence and progression of the disease through multiple mechanisms (Mirzalieva et al., 2022). Studies have shown that ISG15 is involved by marking proteins for degradation, antagonizing protein degradation (stabilizing proteins), affecting protein localization, preventing the formation of protein complexes, or regulating immune responses (Albert et al., 2018). These mechanisms of action may collectively contribute to the immune system disorder and disease progression in SLE patients.

Recent studies have also emphasized the inductive effect of ISG15 on other cytokines, such as CXCL1, CXCL5, tumor necrosis factor (TNF), IL-1, and IL-6 (Álvarez et al., 2024). These cytokines also play an important role in the pathogenesis of SLE. In addition, extracellular ISG15 also acts as a neutrophil chemoattractant, inducing the proliferation of natural killer (NK) cells, stimulating the production of interferon-γ, and inducing the maturation of dendritic cells (Perng and Lenschow, 2018). These findings further enrich our understanding of the mechanism of ISG15’s role in SLE.

In our study, through molecular docking technology, we found that the key gene ISG15 can effectively bind to the two compounds, genistein and Alvocidib. This discovery is of great significance for understanding the mechanism of action of these compounds in anti-inflammatory and immune regulation, especially for autoimmune diseases such as systemic lupus erythematosus (SLE). Genistein, a major isoflavone compound, has been proven to have potentially beneficial effects on a variety of degenerative diseases and chronic illnesses, mainly due to its anti-inflammatory and immunosuppressive properties (Chen et al., 2019). This suggests that it plays an important role in immune system regulation. Further research has shown that genistein can also reduce cell-mediated immunity in animals and decrease delayed-type hypersensitivity (DTH) (Yellayi et al., 2002).

Studies have also found that genistein can not only delay the rejection of rat heart allografts, indicating its immunosuppressive effect *in vivo*, but also reduce the number of developing CD4^+^ and CD8^+^ thymocytes (Ganai and Farooqi, 2015), This reveals a possible mechanism by which it affects cell-mediated immunity. In SLE, abnormal activation of T cells is a key factor leading to the occurrence and development of the disease. Therefore, genistein may have a positive therapeutic effect on SLE by reducing the number or activity of these cells.

On the other hand, Alvocidib, as a first-generation pan-CDK inhibitor, is the first drug to enter clinical trials and be widely studied (Zhang et al., 2021). Animal studies have found that CDK inhibitors, such as Alvocidib, have shown improvement in nephritis in animal models, possibly by reducing the proliferation of lupus T and B cells (Hubbard et al., 2020). In SLE, abnormal proliferation and activation of T and B cells are key factors leading to disease progression. Therefore, Alvocidib has the potential to slow down the condition of SLE by inhibiting the proliferation of these cells. It has been shown to inhibit interferon-γ-mediated inflammatory responses by reducing the expression of inducible nitric oxide synthase (INOS) and a wide range of inflammatory mediator genes (Sangsuwan et al., 2022).

In addition, alvocidib can also inhibit the formation of nitric oxide (NO) in vascular endothelial cells mediated by interferon-γ, and lead to the inactivation of STAT1 in the JAK/STAT signaling pathway and its downstream interferon-γ response factor (IRF1), which further confirms its anti-inflammatory effect (Terashima et al., 2012). Nitric oxide and tumor necrosis factor-alpha are two key inflammatory molecules, and their overproduction is closely related to the pathophysiology of many inflammatory diseases (Sharma et al., 2007). Studies have shown that under specific conditions, alvocidib can inhibit the production of these two molecules, possibly by regulating the MAPK and NF-κB signaling pathways, thus explaining the importance of its anti-inflammatory effect (Joshi et al., 2023). alvocidib can inhibit the production of these molecules, potentially reducing inflammatory responses and tissue damage in SLE patients.

## Conclusion

In this study, through a comprehensive analysis of SLE-related datasets in the GEO database, the pathogenesis of SLE was deeply explored, with particular focus on immune cell infiltration and the role of the key gene ISG15. The study found that ISG15 plays a critical role in SLE, potentially exacerbating inflammatory responses and tissue damage in SLE through various mechanisms such as affecting mitochondrial function, DNA damage response, and enhancing type I interferon reactions. In addition, through molecular docking technology, the study also discovered that ISG15 can effectively bind to genistein and alvocidib, two compounds with anti-inflammatory and immunosuppressive effects, respectively. This provides new potential drug targets for the treatment of SLE. These findings not only enhance our understanding of the pathogenesis of SLE but also provide important clues for developing new treatment strategies.

## Data Availability

The datasets generated and analyzed in this study are available in the GEO data repository (https://www.ncbi.nlm.nih.gov/geo/query/acc.cgi?acc=GSE65391): GSE65391. Additionally, the datasets used for Mendelian randomization in this study can be found in the IEU Open GWAS Project repository, with the exposure ID being prot-a-1573 (https://gwas.mrcieu.ac.uk/datasets/prot-a-1573/) and the outcome ID for systemic lupus erythematosus (SLE) being finn-b-M13_SLE (https://gwas.mrcieu.ac.uk/datasets/finn-b-M13_SLE/).
