# Supplementary figures and images for: Identification of potential therapeutic targets for systemic lupus erythematosus based on GEO database analysis and Mendelian randomization analysis
Source: Front Genet. 2024 Oct 16;15:1454486. doi: 10.3389/fgene.2024.1454486 (PMC11496559; doi:10.3389/fgene.2024.1454486)

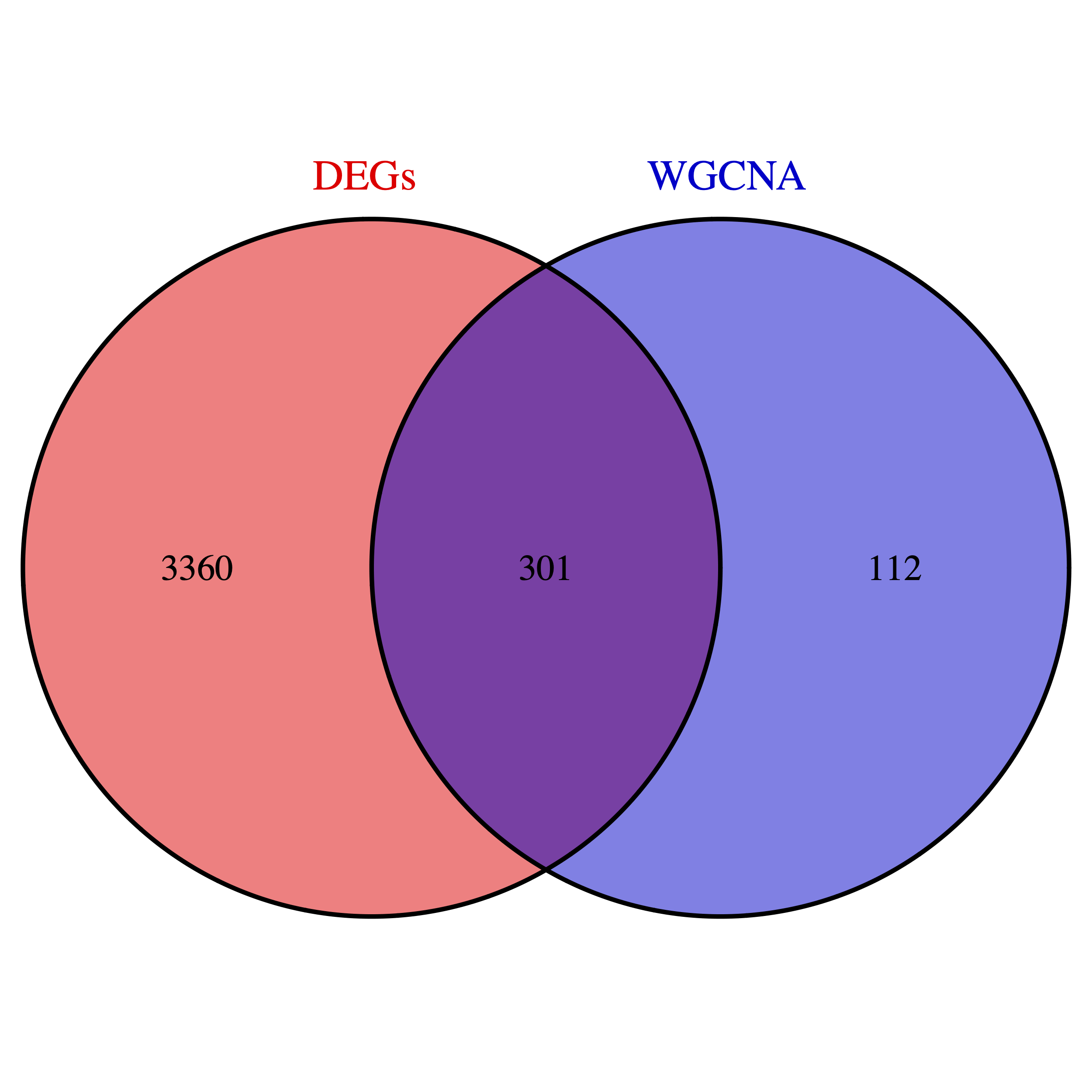

Supplement: Supplementary file 1 [file Image5.TIFF]

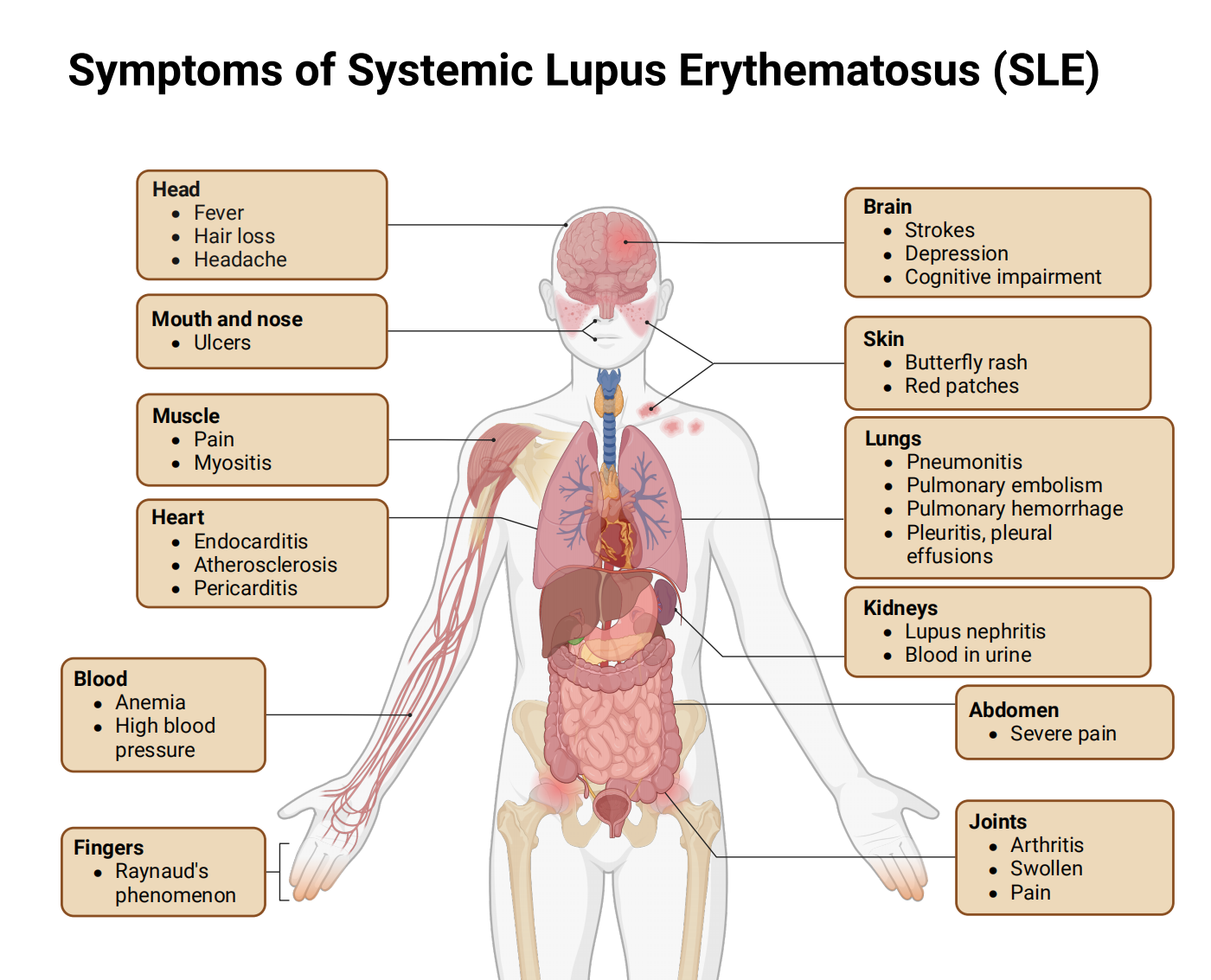

Supplement: Supplementary file 2 [file Image1.tif]

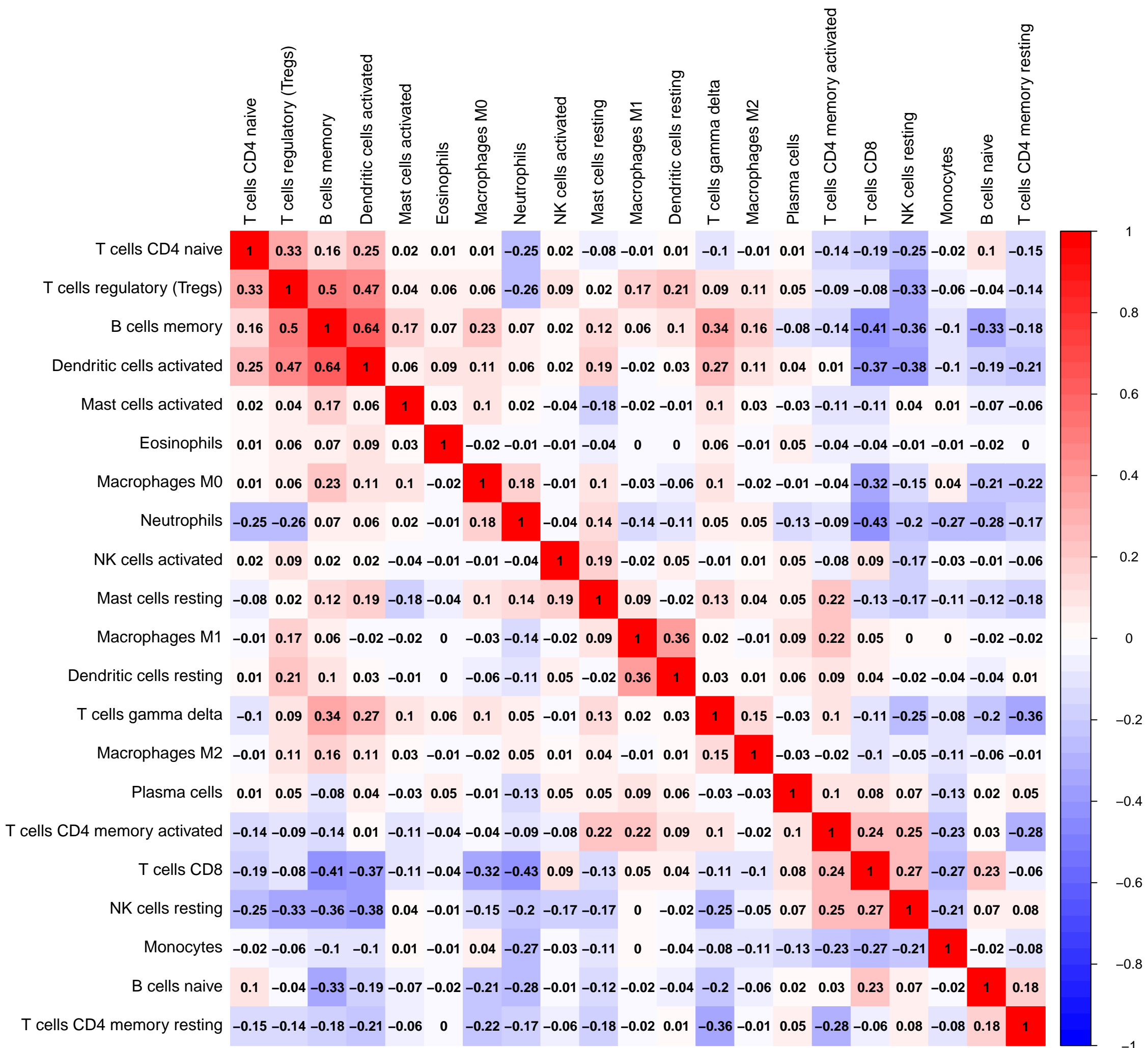

Supplement: Supplementary file 3 [file Image4.pdf]

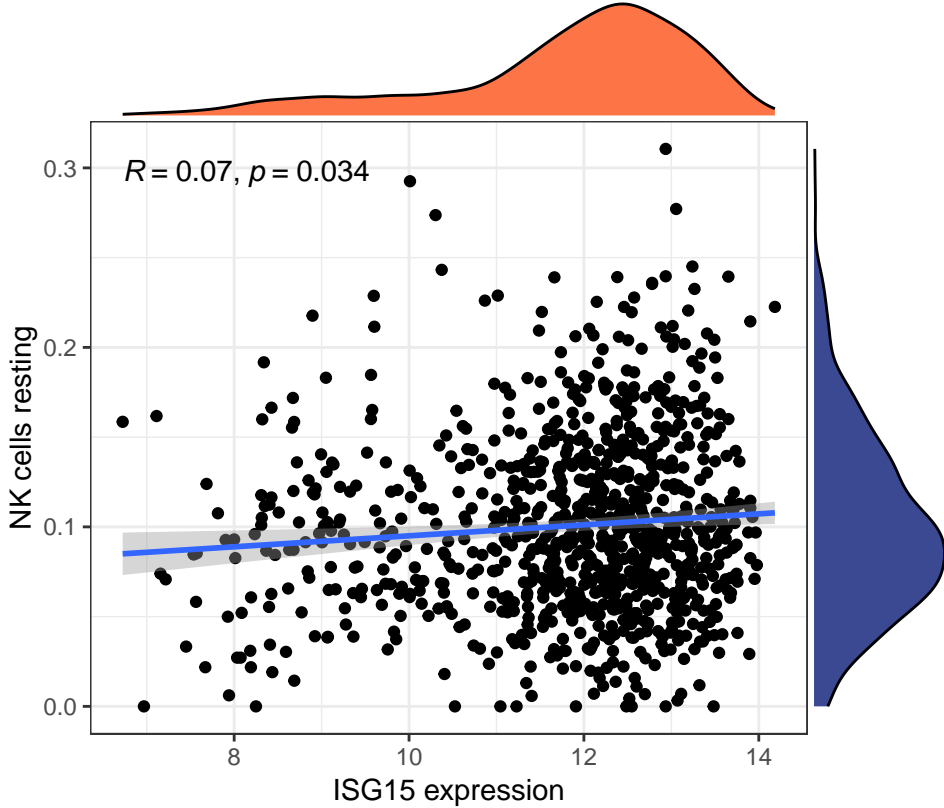

Supplement: Supplementary file 4 [file Image2.pdf]

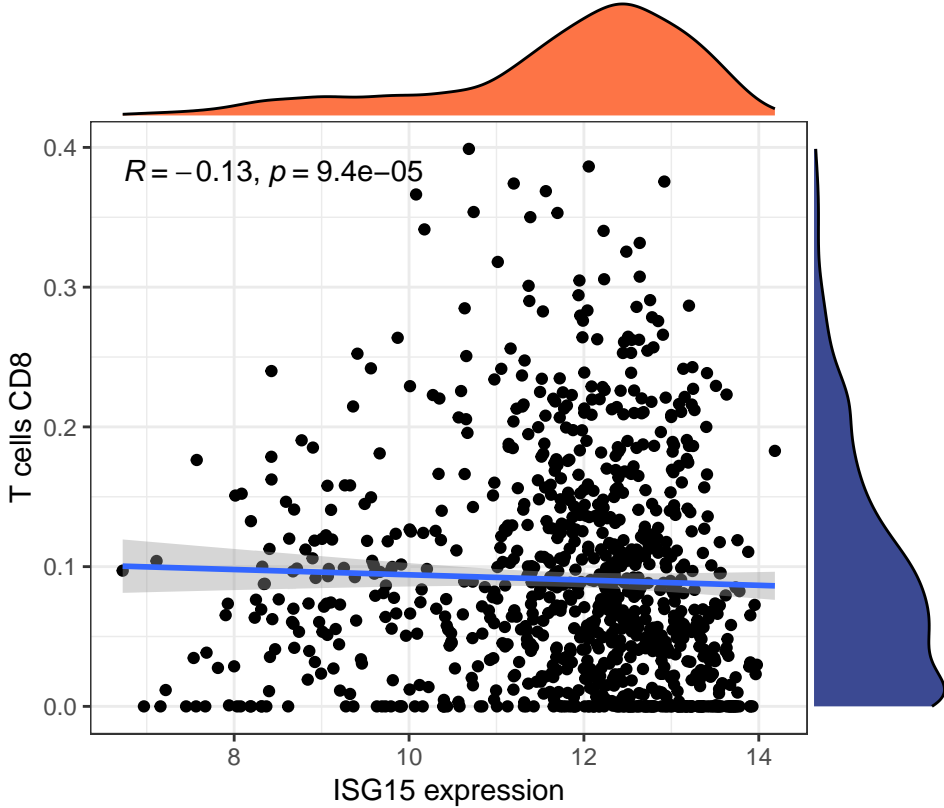

Supplement: Supplementary file 5 [file Image3.pdf]

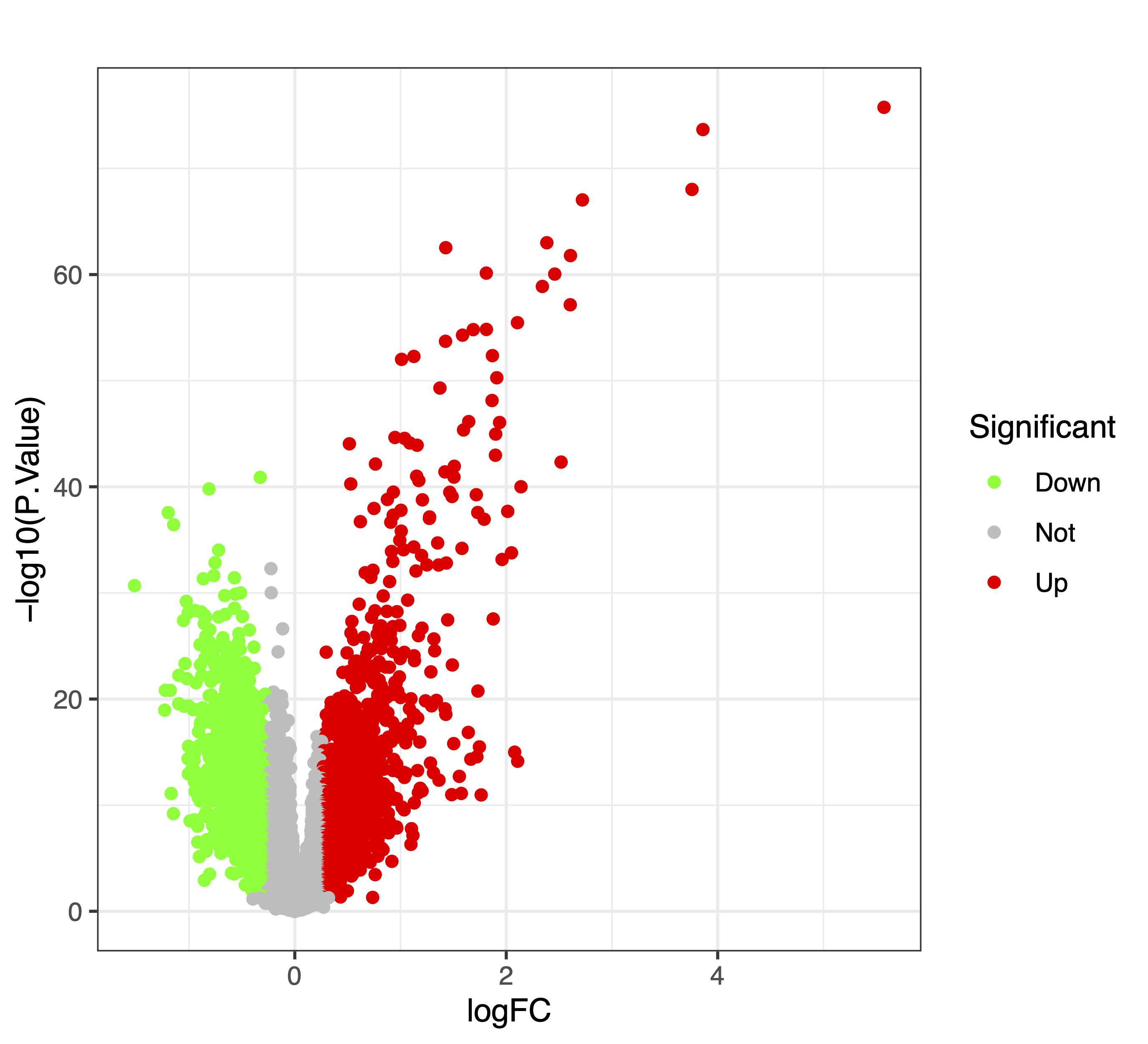

Supplement: Supplementary file 6 [file Image6.TIFF]
